# Sero-Surveillance to Evaluate Trends in the Transmission of SARS-CoV-2 in a Central Indian District

**DOI:** 10.7759/cureus.78843

**Published:** 2025-02-11

**Authors:** Pragati G Rathod, Sarita K Sharma, Ajaya Krishnan P, Thungamithirai Prakash, Uday Narlawar, Surya Kannan, Ekansha Tabhane

**Affiliations:** 1 Department of Community Medicine, Government Medical College and Hospital, Nagpur, Nagpur, IND; 2 Department of Independent Research, McNair Academic High School, Jersey City, USA

**Keywords:** covid-19, general population, sars-cov-2, seroprevalence, serosurvey, transmission trends

## Abstract

Background and objective

The actual community burden of SARS-CoV-2 is undervalued, as the estimates are just the symptomatic infections. The acute phase of the pandemic has waned, and the analytical comparison of infection spread through repeated sero-epidemiological studies is important in the formulation of effective public health strategies. This study investigated the level of seroprevalence of IgG antibodies for the SARS-CoV-2 virus in the Nagpur district, Maharashtra, India.

Materials and methods

The present cross-sectional survey was conducted over three months from September to November 2021 by carrying out a door-to-door survey involving 6129 participants. Among them, 3131 were from municipal areas, while the rest were from non-municipal regions. Data collection was facilitated using Google Forms (Google LLC, Mountain View, California, United States). Venous blood samples were collected, and SARS-CoV-2 antibodies were detected using the COVID KAVACH IgG enzyme-linked immunosorbent assay (ELISA) kit (developed by the National Institute of Virology (NIV), Pune, India). The information collected was then cleaned, coded, and analyzed using Epi Info software (Centers for Disease Control and Prevention (CDC), Atlanta, Georgia, USA).

Results

The seroprevalence rate was found to be 80% in the whole district. Females had a higher seroprevalence rate in both areas than males. The population aged 60 years and above had the highest seroprevalence rate in both zones. The vaccinated group demonstrated a greater number of individuals testing positive for SARS-CoV-2 compared to the unvaccinated group.

Conclusion

The significant increase in the seroprevalence estimates in relation to the prior survey is because of the significant surge in COVID-19 vaccination coverage after the first wave of the pandemic. The findings of the study imply the dynamic nature of the pandemic and the different degrees of immunity obtained within the community. Ongoing surveillance and research are essential for refining effective strategies to manage and mitigate future pandemics effectively.

## Introduction

The COVID-19 pandemic has profoundly moved the world to the edge since its emergence in December 2019. The pandemic has wreaked health systems equally in both developed and developing nations around the globe, leading to significant global mortality and morbidity [1]. According to the Ministry of Health and Family Welfare (MoHFW), Government of India, the total number of COVID-19 cases in India was more than 44 million, with a mortality rate of 1.19% [2].

The acute phase of the pandemic has subsided, but understanding the actual reach of virus spread is still of paramount importance. The reported cases often underestimate the actual community burden due to factors such as asymptomatic infections, variations in testing strategies over time and locations, different sensitivities of diagnostic tests, and health-seeking behaviors [1]. Further, the dynamics of transmission have been heterogeneous, resulting in differences in seroprevalence rates among populations [3]. The World Health Organization (WHO) and other agencies have suggested carrying out population-based serosurveys to address this issue [4]. There has also been a standardized protocol for these studies devised by WHO to make the data collection process rigorously systematic and data sharing as fast as possible, thus facilitating comprehensive analysis [1].

The overall seroprevalence (i.e., the proportion of the population with SARS-CoV-2 antibodies) estimated from a previous study, conducted from October to November 2020 in Nagpur district, turned out to be 35.17%. The seropositivity rate in the municipal areas was 49.7%, more than twice that of the non-municipal area (20.7%) [5]. Repeated cross-sectional sero-epidemiological studies are vital for monitoring the trends of disease over time, guiding infection prevention, and preparing control strategies through effective public health interventions [6].

Elaborating on this, a second round of serosurvey was planned to evaluate the seroprevalence of IgG antibodies to SARS-CoV-2 infection in the Nagpur district of Maharashtra, Central India, thus analyzing the spread of the virus in the population, so as to design and implement appropriate health system and policy-level interventions for future pandemic preparedness. The present study will provide a historical baseline for future serosurveys that can be used for comparative analysis to evaluate trends in immunity and transmission. By integrating newer seroprevalence data with our findings, public health authorities can better understand immunity gaps, optimize booster vaccination policies, and refine strategies for managing future outbreaks.

## Materials and methods

Study design, duration setting, and participants

The present cross-sectional study was carried out at Government Medical College, Nagpur, in the Nagpur district of Maharashtra, located in central India, over a period of three months from September to November 2021. The study area was divided into Nagpur Municipal Corporation (NMC) and non-NMC areas.

Sample size calculation and sampling

Considering a prevalence rate of 67% [1], with a relative precision of 2.5%, a 95% confidence interval, and a design effect of 2, the sample size was calculated to be 6,054 individuals using Epi Info 7.2.0.1 (Centers for Disease Control and Prevention (CDC), Atlanta, Georgia, USA) and was rounded to 6,100. The district was divided into two regions for the serosurvey: areas under the Nagpur Municipal Corporation (NMC) and non-NMC regions. The Nagpur Municipal Area is administratively divided into 10 zones, while the non-NMC region is divided into 13 talukas. For the assessment, 40 clusters were randomly selected from the NMC zones, with four clusters from each of the 10 zones. In the non-NMC region, 40 clusters were selected from the 13 talukas: for 12 talukas, three clusters each (two from rural areas and one from taluka headquarters), and the remaining taluka, four clusters from rural areas.

**Figure 1:**
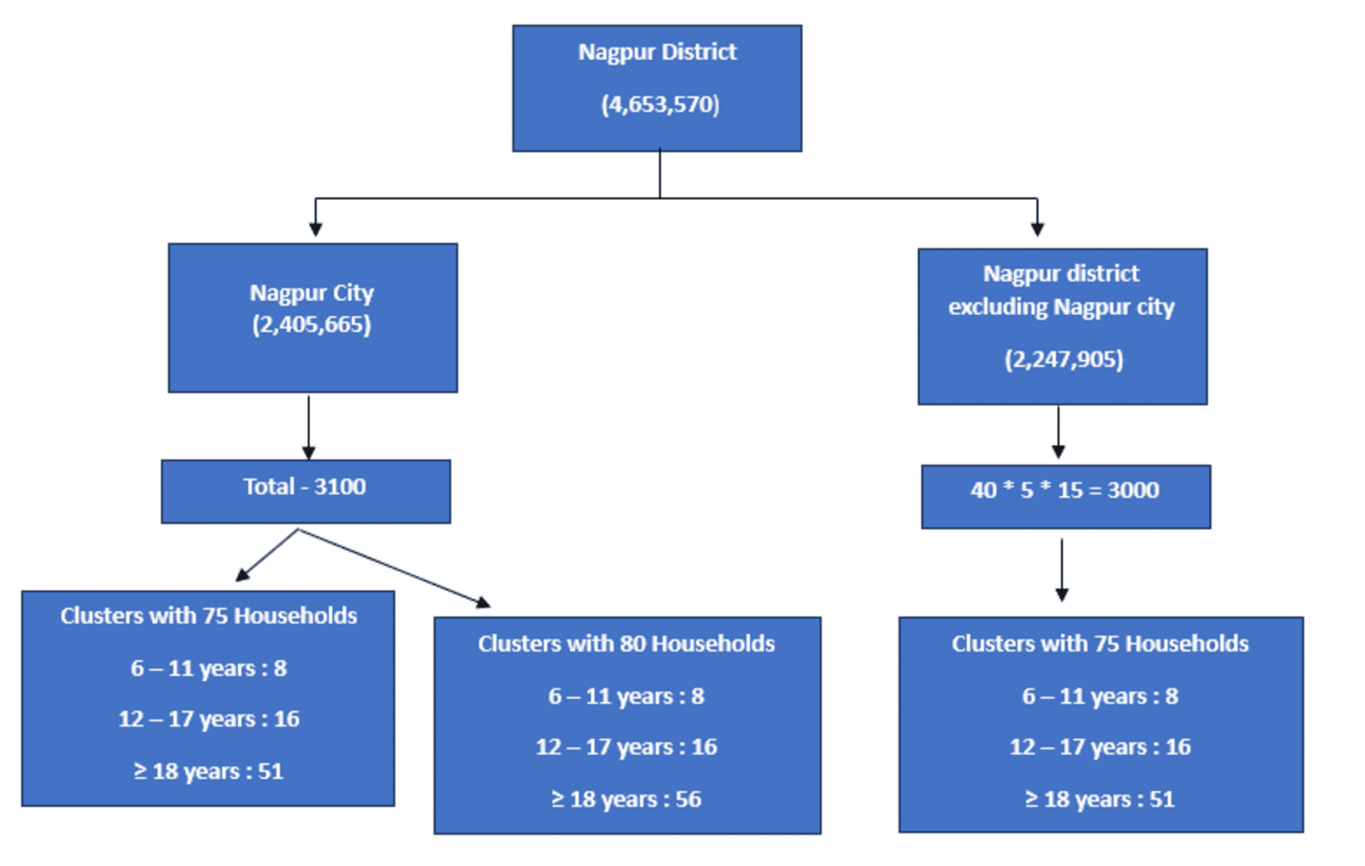
Flowchart for selection of clusters and households.

To ensure a representative sample, five entry points were identified in each selected cluster. In the NMC areas, one individual from 15 consecutive households was surveyed at each entry point in 20 clusters, and one individual from 16 consecutive households in the remaining 20 clusters, to give a total sample size of 3,100. Thus, there were two types of clusters, one comprising 75 households and the other comprising 80 households. For both cluster types, eight participants aged six to 11 and 16 participants aged 12-17 were enrolled. Additionally, in clusters with 75 households, 51 participants over the age of 18 were included, while in the 80 household clusters, 56 participants from the same age group were enrolled. Similarly, in the non-NMC areas, one individual from 15 consecutive households at each of the five entry points was surveyed across 40 clusters, resulting in a total of 3,000 samples. In this region, participant enrollment mirrored that of the 75 household clusters in NMC areas (Figure 1).

Study procedure

Approval for the study was obtained from the Institutional Ethics Committee, Government Medical College, Nagpur. Each study team consisted of a medical officer, nursing staff, and a lab technician, all of whom received thorough training in data collection and other necessary procedures. Data collection was supervised by the Department of Health of NMC, Nagpur for the NMC areas and by Zilla Parishad, Nagpur for the non-NMC areas.

The study teams visited the selected households, explained the nature and purpose of the study, and obtained written informed consent from adult participants and assent from parents of children aged six to 12 years. Both consent and assent were taken from the child and his parents, respectively, in the case of participants aged 13 to 17 years. As outlined in Table 1, one consenting individual from each selected household was included in the study as per the schema [7]. For instance, if a household consisted of three adults, with one being female, the oldest male was selected to participate in the study.

**Table 1:** Schema for selection of individuals from selected households in the cluster.

Number of adult females in the household	Number of adults in the household			
	1	2	3	4
0	Male	Youngest male	Youngest male	Oldest male
1	Female	Female	Oldest male	Female
2		Oldest female	Male	Oldest male
3			Youngest female	Older male
4 or more				Oldest female

Households were excluded if any members had tested positive for SARS-CoV-2 by reverse transcription-polymerase chain reaction (RT-PCR) or if they refused to provide consent for participation. If no eligible individual was available in a household, the team proceeded to the next household until the required sample size was achieved. Basic demographic information, history of exposure to laboratory-confirmed COVID-19 cases, symptoms suggestive of COVID-19, and history of comorbidities such as diabetes and hypertension in adult participants were collected using Google Forms (Google LLC, Mountain View, California, United States). Following the submission of Google Forms, a blood sample of 3-5 ml was collected from each eligible participant, ensuring all necessary precautions. These samples were then transported to the laboratory at Government Medical College, Nagpur, maintaining a cold chain.

In the laboratory, the sera were tested for IgG antibodies using the COVID KAVACH enzyme-linked immunosorbent assay (ELISA) kit developed by the National Institute of Virology (NIV), Pune. The COVID KAVACH ELISA kit is the first indigenous anti-SARS-CoV-2 human IgG ELISA test kit for detecting SARS-CoV-2 antibodies [8]. It offers a quicker and easier alternative to conventional RT-PCR tests, making it suitable for assessing a large number of samples, such as those in surveillance studies.

Statistical analysis

The data collected through Google Forms were analyzed using Epi Info 7.2.0.1 software. Descriptive statistics, including percentages and summary measures, were used to describe the characteristics of the study participants. The unadjusted seroprevalence of COVID-19 IgG antibodies was reported as a percentage with a 95% confidence interval (CI). The chi-square test was employed to analyze the association between various categories and the seroprevalence rate, with a p-value of less than 0.05 considered statistically significant. A Z-test for proportions was applied to compare the community seroprevalence rates post-COVID-19 wave 1 and wave 2.

## Results

In the NMC area, the coverage exceeded 100%, resulting in a total sample of 3,131, whereas, in the non-NMC area, the coverage was 99.93%, leading to a total sample of 2,998 samples. Hence, the total sample size from both the areas taken together comprised 6,129 subjects. The study population constituted 3,646 females (59.49%) and 2,483 males (40.51%), the sex ratio being 1468:1000. Out of the 3,131 study participants from the NMC area, 1,115 (44.90%) were males and 2,016 (55.30%) were females. In contrast, among the 2,998 study subjects from the non-NMC area, 1,630 (54.40%) were females and the rest were males. The seroprevalence rates in NMC and non-NMC zones were found to be 84% (95% CI: 82.67 - 85.24%) and 75.91% (95% CI: 74.35 - 77.41%), respectively. The overall seroprevalence rate was determined to be 80.05% (95% CI: 79.03-81.03%).

Table 2 presents the detailed seroprevalence rates across different categories.

**Table 2:** Distribution of seroprevalence among the study participants. ^*^ statistically significant (p < 0.05); NMC: Nagpur Municipal Corporation

Variable	Total tested	Total reactive	Percentage (%) (95% CI)	Chi-square; p-value
Place of Residence				308.43; <0.001^*^
NMC	3131	2630	84.00 (82.67 – 85.24)	
Non-NMC	2998	2276	75.91 (74.35 – 77.41)	
Total	6129	4906	80.05 (79.03 – 81.03)	
Gender				5.971; 0.014^*^
Male	2483	1950	78.53 (76.88 – 80.10)	
Female	3646	2956	81.08 (79.77 – 82.31)	
Age group (in years)				3.99; 0.406
6 – 11	892	706	79.15 (76.48 – 81.81)	
12 – 17	736	597	81.11 (78.29 – 83.94)	
18 – 45	2730	2164	79.27 (77.75 – 80.79)	
46 – 60	1125	919	81.69 (79.43 – 83.95)	
> 60	646	520	80.50 (77.44 – 83.55)	

**Figure 2:**
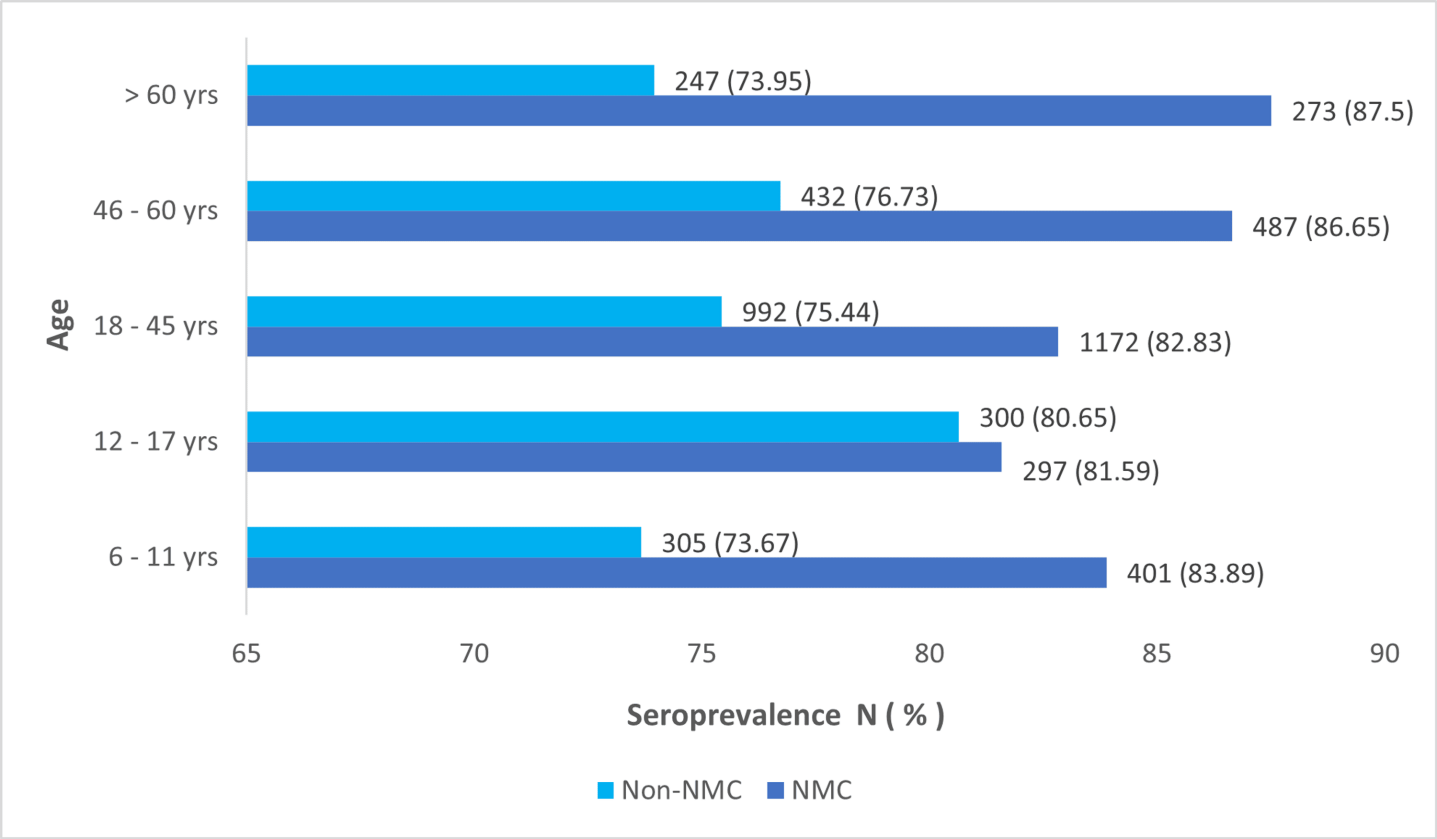
Age-wise distribution of seroprevalence in NMC and non-NMC zones. NMC: Nagpur Municipal Corporation

The highest seropositivity rates were observed among the age group above 60 years in the NMC area, whereas in the non-NMC area, it was found in the 12-to-17-year age group. Figure 2 illustrates the detailed age-wise distribution of seroprevalence.

**Figure 3:**
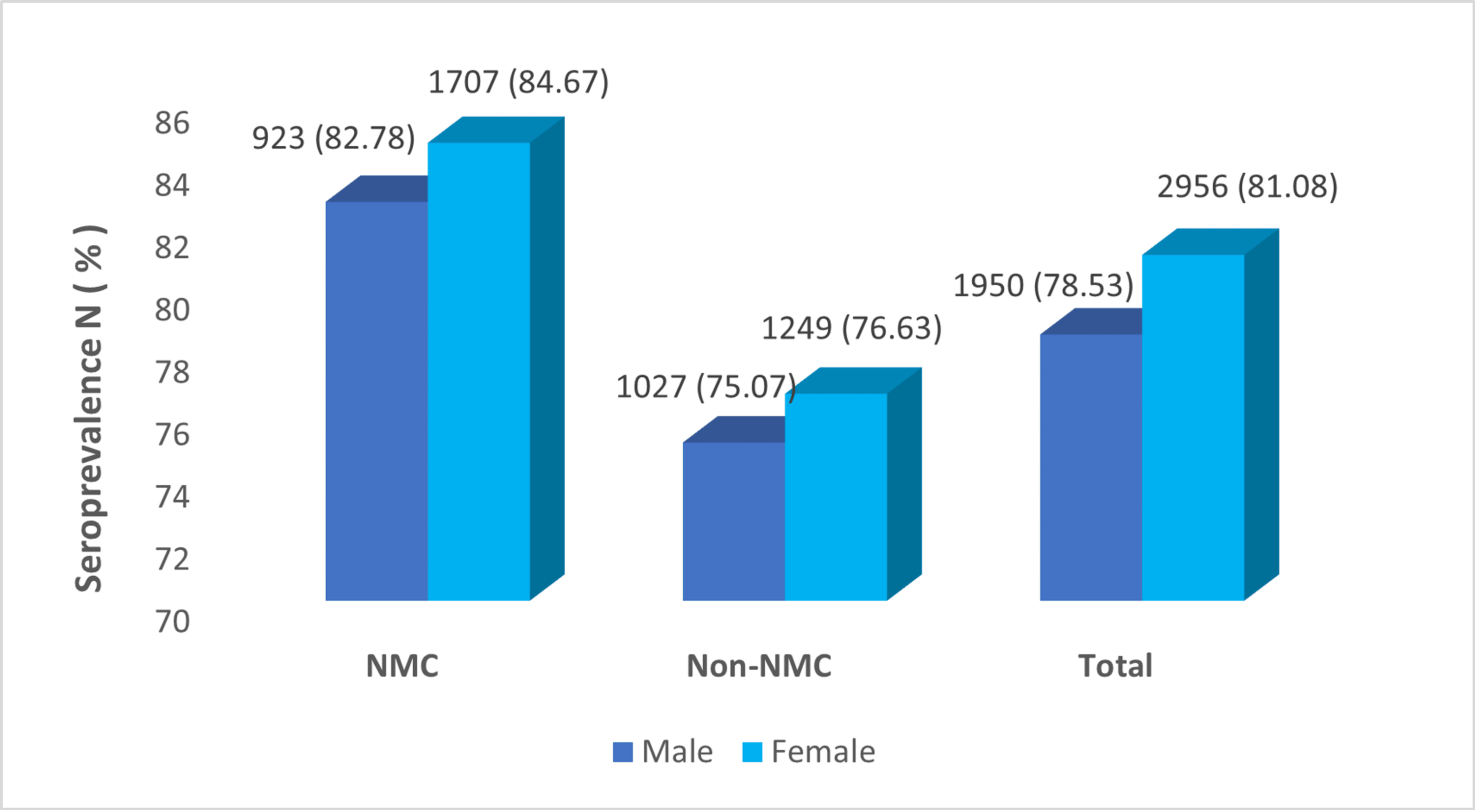
Gender-wise distribution of seroprevalence in NMC and non-NMC zones. NMC: Nagpur Municipal Corporation

Seroprevalence was higher among females than males in both NMC and non-NMC zones. Gender-wise distribution of seroprevalence in both zones is shown in Figure 3. 

The most common comorbid conditions present among the study participants were hypertension (250, 4.07%), followed by diabetes mellitus (184, 3%). Among the hypertensive, 238 (95.2%) were found seropositive, while 156 (84.78%) of diabetics were seropositive in the survey.

Regarding vaccination status for COVID-19 in this study, from the NMC area, only 682 individuals (21.78%) had received both doses, while 548 individuals (17.50%) had received only the first dose. whereas in the non-NMC area, 1,222 participants (40.76%) had received both doses, and 708 participants (23.6%) had received only the first dose. During data collection, 137 individuals (4.37%) in the NMC zones and 339 individuals (11.31%) in the non-NMC zones reported experiencing COVID-19-related symptoms. Additionally, 221 participants (7.05%) in the NMC zones and 569 participants (18.98%) in the non-NMC zones reported close contact with COVID-19 patients within the last 14 days.

Significant differences were observed in the distribution of COVID-19 symptoms, contact history with confirmed COVID-19 cases, and vaccination status between urban NMC zones and rural non-NMC zones. A notably higher proportion of individuals in non-NMC zones had received two vaccine doses, reported COVID-19-related symptoms, and had a history of contact with confirmed cases compared to those in municipal areas (p < 0.05).

The sero-reactivity assessed using the KAVACH ELISA kit showed a statistically significant association with the presence of COVID-19-related symptoms and a history of contact with confirmed COVID-19 cases within the NMC and non-NMC zones, respectively. However, when all the study participants were considered, no significant association was observed between the seroprevalence rate and these variables (p ≥ 0.05). Although the seroprevalence rate was higher among individuals who had received two doses of the COVID-19 vaccine, the association was found not statistically significant (p ≥ 0.05). The distribution of seroprevalence among study participants based on these categories is provided in Table 3.

**Table 3:** Seroprevalence distribution based on vaccination status, history of close contact, and presence of COVID-19 symptoms. NMC: Nagpur Municipal Corporation

Categories	NMC (n = 3131)			Non-NMC (n = 2998)			Total (N = 6129)		
	Total tested	Total reactive	Percentage (95% CI)	Total tested	Total reactive	Percentage (95% CI)	Total tested	Total reactive	Percentage (95% CI)
Symptoms									
Present	137	127	92.70 (88.34 – 97.06)	339	263	77.58 (73.14 – 82.02)	476	390	81.93 (78.48 – 85.39)
Absent	2994	2503	83.6 (82.27 – 84.93)	2659	2013	75.71 (74.08 – 77.34)	5653	4516	79.89 (78.84 – 80.93)
Contact with COVID-19 patients									
Yes	221	177	80.09 (74.83 – 85.36)	569	473	83.13 (80.05 – 86.21)	790	650	82.28 (79.62 – 84.94)
No	2215	1895	85.55 (84.09 – 87.02)	1849	1362	73.66 (71.65 – 75.67)	4064	3257	80.14 (78.92 – 81.37)
Don’t Know	695	558	80.28 (77.33 – 83.25)	580	441	76.03 (72.56 – 79.51)	1275	999	78.35 (76.09 – 80.61)
Received COVID-19 vaccination									
1 dose	548	458	83.57 (80.47 – 86.68)	708	549	77.54 (74.47 – 80.62)	1256	1007	80.18 (77.97 – 82.38)
2 doses	682	571	83.72 (80.95 – 86.49)	1222	926	75.78 (73.38 – 78.18)	1904	1497	78.62 (76.78 – 80.47)

**Figure 4:**
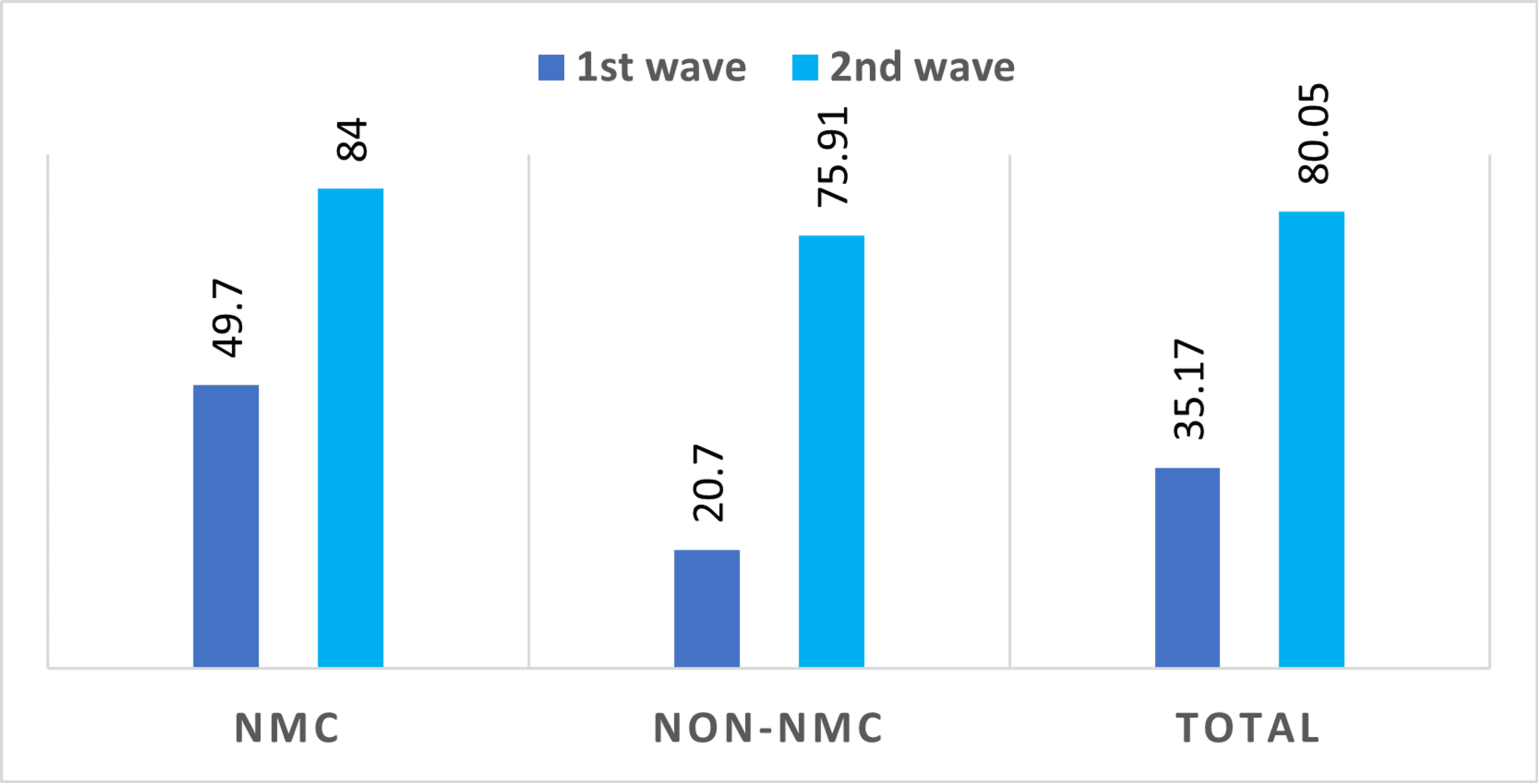
Comparison of seroprevalence rate post-COVID-19 wave 1 and 2. NMC: Nagpur Municipal Corporation

In the present seroprevalence survey from Nagpur district, the overall seroprevalence rate was 80.05%, indicating that a major portion of the population is exposed to the COVID-19 virus either through infection or vaccination. The previous phase 1 serosurvey conducted post-COVID-19 first wave demonstrated a total seroprevalence of 49.7% in the district [5]. A comparison of the seroprevalence rate in the district post wave 1 and 2 of COVID-19 is provided in Figure 4. A significant difference in seroprevalence rates was observed post wave 1 and wave 2, both individually within municipal and non-municipal areas and across the whole district (p < 0.05).

## Discussion

By the end of the current survey, Nagpur recorded nearly 364,048 cases, including 6,053 deaths. Subsequently, similar to the nationwide pattern, the increase in the seroprevalence rate as compared to post-COVID-19 wave 1 was due to both natural infection as well as COVID-19 vaccination.

A 2021 study by Sharma P et al. [6] in Delhi observed similar trends to those in our current research, reporting a high seroprevalence rate. Their study found an 89.5% seroprevalence, compared to our study's 80%. Notably, both studies were conducted around the same time frame. A meta-analysis of seroprevalence studies carried out in India [1] during the first and second waves revealed a pooled seroprevalence rate of 69% in the country following the second wave.

Consistent with the findings of Sharma P et al. [6], our study also observed a higher seroprevalence rate among females compared to males. According to a multicentric seroprevalence survey conducted by ICMR [9] up to July 2021, the seroprevalence rates were 52% for females and 48% for males. The elevated seroprevalence rates in both genders in our study may be attributed to it being conducted a few months after the aforementioned study.

The seroprevalence rate was highest among individuals over 60 years of age and children as compared to other age groups. Similar findings were observed in studies [10-14] conducted during both the first and second waves of SARS-CoV-2 in India, indicating that the elderly population was more susceptible to acquiring the infection throughout all waves. Due to the exposure at the household level to other members, children and adults over the age of 60 years show greater seropositivity as these family members are socially more active, have greater mobility, and comply less with the recommended infection prevention and control measures.

In line with the ICMR pan-Indian study [9], our research also reveals no significant difference in seroprevalence rates between NMC (urban) and non-NMC areas. However, there is a slightly higher prevalence among the NMC population compared to their non-NMC counterparts.

A study conducted among contacts of SARS-CoV-2 in Gujarat and Delhi [13,15] after the first wave reported a seropositivity rate of 30%. In contrast, our current study, conducted after the second wave, shows a significantly higher seroprevalence rate of nearly 80%. This increase can be attributed to the sharp rise in SARS-CoV-2 infections during the second wave nationwide, as well as the rapid surge in COVID-19 vaccinations following the first wave. Similar findings have been reported in multiple studies conducted worldwide, linking the increase in seroprevalence rates to the impact of vaccination programs and the dynamics of disease transmission [16-21].

This study possesses several notable strengths. A large sample size of 6129 participants strengthens the reliability and generalizability of the study findings to a larger population. The geographic diversity covering both municipal and non-municipal areas ensures the study has a greater external validity. The use of a door-to-door method of data collection reduces selection bias and increases the chances of better participation, resulting in more accurate seroprevalence estimates. Including a wide age range, from children above six years to older adults, allows for a comprehensive assessment of immunity across different age categories.

This study has certain inherent limitations. COVID-19 seroprevalence was assessed using an ELISA kit instead of the more accurate RT-PCR test. Antibody levels may diminish over time, and only one individual per household was included in the study due to feasibility issues, potentially leading to an underestimation of seroprevalence given the transmission dynamics within households. These factors might have influenced the ability to accurately determine the true seropositivity rate in the community. Additionally, while the unique nature of the disease might encourage participants to recall and report symptoms, there remains a possibility of recall bias.

## Conclusions

In conclusion, the present study offers substantial evidence regarding the prevalence of SARS-CoV-2 antibodies among the general community in a district in Central India, concluding the presence of SARS-CoV-2 IgG antibodies among the vast majority of the population in the district. The marked increase in the seroprevalence rates as compared to the previous survey can be explained by the remarkable increase in COVID-19 vaccination coverage among the general population following the first wave of the pandemic. At this moment, the findings of the study imply the dynamic nature of the pandemic and the different degrees of immunity obtained within the community. The findings are of importance in the development of fruitful public health policies and vaccination campaigns and can also guide the succeeding research to help prevent future pandemics like COVID-19 and minimize the devastating effects those diseases can have. Ongoing surveillance and continued research are vital to manage and mitigate the impact future pandemics can have on mankind.
